# Teaching Cognitive Behavior Therapy to Postgraduate Health Care Professionals in Times of COVID 19 – An Asynchronous Blended Learning Environment Proved to Be Non-inferior to In-Person Training

**DOI:** 10.3389/fpsyg.2021.657234

**Published:** 2021-09-27

**Authors:** Daniel Soll, Raphael Fuchs, Stephanie Mehl

**Affiliations:** ^1^Department of Psychiatry and Psychotherapy & Center for Mind, Brain and Behavior (CMBB), Faculty of Medicine, Philipps-University Marburg, Marburg, Germany; ^2^Department of Health and Social Work, Frankfurt University of Applied Sciences, Frankfurt am Main, Germany

**Keywords:** cognitive behavior therapy (CBT), therapist training, inverted classroom, online training, blended learning, non-inferiority

## Abstract

Training of postgraduate health professionals on their way to becoming licensed therapists for Cognitive Behavior Therapy (CBT) came to a halt in Germany in March 2020 when social distancing regulations came into effect. Since the German healthcare system almost exclusively relies on this profession when it comes to the implementation of CBT and 80% of those therapists active in 2010 will have retired at the end of 2030, it is critical to assess whether online CBT training is as satisfactory as classroom on-site CBT training. An asynchronous, blended, inverted-classroom online learning environment for CBT training (CBT for psychosis) was developed as an emergency solution. It consisted of pre-recorded CBT video lectures, exercises to train interventions in online role-plays, and regular web conferences. Training was provided at five different training institutes in Germany (duration 8–16 h). Postgraduate health care professionals (psychiatrists and psychologists) (*n* = 43) who received the online CBT training filled out standard self-report evaluations that assessed satisfaction and didactic quality. These evaluations were compared to those evaluations of students (*n* = 142) who had received in-person CBT training with identical content offered by the same CBT trainer at the same training institutes before the COVID-19 crisis. Both groups were comparable with respect to *interest in the subject* and *prior knowledge*. We tested non-inferiority hypotheses using Wilcoxon-Mann-Whitney ROC-curve analyses with an equivalence margin corresponding to a small-to-medium effect size (*d* = 0.35). The online training evaluations were non-inferior concerning *information content, conception of content, didactic presentation, assessment of the trainer as a suitable role-model, working atmosphere, own commitment*, and *practical relevance*. In contrast, we could not exclude a small effect in favor of in-person training in *professional benefit* and *room for active participation*. Our results suggest that delivering substantial CBT knowledge online to postgraduate health-professionals is sufficient, and at most incurs minimal loss to the learning experience. These encouraging findings indicate that integrating online elements in CBT teaching is an acceptable option even beyond social distancing requirements.

## Introduction

Cognitive Behavior Therapy (CBT) is effective across a wide range of mental disorders, e.g., depression (Cuijpers et al., 2013), psychotic disorders (Bighelli et al., 2018), and anxiety disorders (Cuijpers et al., 2016). With regard to psychosis, CBT has been recommended by several national guidelines [Germany: (German Association for Psychiatry, Psychotherapy and Psychosomatics (DGPPN), 2019); United Kingdom: (National Institute for Health and Clinical Excellence (NICE), 2019)]. However, in most countries, there are still implementation problems [e.g., in Germany (Schlier and Lincoln, 2016)], often due to a lack of therapists’ training (Heibach et al., 2014).

Best practice elements of CBT training have been identified in a review based on 35 randomized-controlled CBT trials (Rakovshik and McManus, 2010). Based on this review, a combination of theoretical instructions with experiential and interactive training elements (reflection on practice cases and role-play) with ongoing regular supervision is considered the “gold standard.” The inclusion of experiential and interactive training elements has been proven effective for delivering theoretical knowledge and improvement of therapeutic attitudes and behavior, which in turn leads to improved CBT outcomes (Beidas and Kendall, 2010).

Postgraduate CBT training in Germany does meet this gold standard and is provided by more than 230 institutes (Psychotherapeutenvereinigung, 2020). Students are both medical and psychological graduates. The mandatory requirement of 600 h of theoretical training courses is delivered by licensed and experienced CBT therapists in small groups (≤18 students) and usually includes lectures and experiential and interactive elements. CBT training is completed with a state examination and is regulated by state law (PsychTHG, 1998).

The German health care system relies heavily on regular enrollment of trained CBT therapists, as every patient diagnosed with a mental disorder according to the International Classification of Diseases (ICD-10; Dilling and Mombour, 2015) qualifies for therapy free of charge (25–80 sessions based on individual need). The ongoing demographic change makes this an even more pressing matter as 80% of the present CBT therapists will have been retired at the end of 2030 (Nübling et al., 2010).

At the beginning of the COVID-19 crisis in March 2020, Germany’s theoretical training courses came to a sudden halt as state laws prohibited gathering in groups. In response, most CBT training institutes and trainers broke new ground by switching to online teaching.

Concerning online learning/e-learning, there is a tremendous variety of different approaches, one distinctive feature of these approaches being the synchronicity of presentation and reception. Synchronous online courses are often virtual classrooms modeled similar to in-person-classrooms: lectures are directly held in web conferences, and training sessions are performed in the virtual classroom. Synchronous courses have some advantages, e.g., they are more familiar for learners, but also disadvantages, e.g., all participants have identical schedules, which might be especially hindering whenever different time-zones are involved. Besides, long web conferences may be somewhat stressful, as they make it harder for participants to perceive additional information such as non-verbal cues. This stress often leads to the currently discussed “Zoom fatigue” (Wiederhold, 2020).

In the purest form of asynchronous training, participants view video-lectures or read texts and train skills independently. Sometimes additional written tasks and quizzes are added to solidify knowledge gain. Participants can learn at an individual learning pace. Still, asynchronous online training often relies too heavily on self-learning and self-monitoring abilities that participants might not always have. Also, participants sometimes feel less connected with their classes and miss the sense of community (Anderson, 2008).

An exciting combination of online and in-person learning is “blended” learning that combines “face-to-face instructions with computer-mediated instructions” (Graham, 2006). One variation of blended learning that includes asynchronous and synchronous elements is the inverted-classroom method (Lage et al., 2000; Handke, 2012): a self-directed learning phase is performed prior to the regular classroom appointment. Students read a lecture or view an instructional screencast. The subsequent classroom appointment is used to answer questions on the content, to reflect on the gained knowledge, to transfer it to practice cases, or to train the newly acquired skills. The most crucial aim of the inverted-classroom method is to enhance the acquisition of more complex skills (analysis, synthesis, and evaluation) during classroom time. In contrast, the self-learning phase is used for lower cognitive processes (acquisition of knowledge) (Tolks et al., 2016).

Thus, this method seems to be a good fit for CBT training, as it involves both synchronous and asynchronous elements. In a recent meta-analysis on studies comparing in-person instructions and inverted-classroom methods in medical education, the inverted-classroom method was superior to traditional methods and associated with more significant academic achievement (examination scores) (Chen et al., 2018). Similar results were obtained in a meta-analysis assessing inverted-classroom methods vs. in-person methods in general academic education (van Alten et al., 2019). Interestingly, while courses presented in inverted-classroom arrangements are superior regarding performance of the enrolled students, students do not rate these courses as more satisfying and acceptable in comparison to courses held in traditional classroom formats (Roehling et al., 2017; van Alten et al., 2019).

With regard to CBT training, there is a consensus that online formats could improve the dissemination of CBT (Shafran et al., 2009; Fairburn and Cooper, 2011) and are an adequate alternative to in-person courses with regard to enhancing participants’ knowledge of the interventions as well as their skills as therapists (Sholomskas et al., 2005; Martino et al., 2011).

Nevertheless, only a small number of studies compared CBT online training courses and in-person courses directly. Stein et al. (2015) compared health care professionals who received an asynchronous self-learning online training (12 h) and an in-person-training (24 h) with respect to the regular application of strategies the professionals learned in both courses (and additional regular supervision). Results revealed that both courses were comparable with regard to the implementation of CBT strategies (notably as rated by therapists’ patients). Similar results were obtained in a direct comparison of synchronous CBT online training that was offered via an avatar on the platform Second Life and in-person training (Mallonee et al., 2018). Though differences between in-person and avatar training with respect to participants’ satisfaction were statistically significant, a vast majority (>90%) of participants were “satisfied “or “very satisfied” in both courses (Mallonee et al., 2018).

In summary, studies suggest that CBT online training courses might be as acceptable and satisfying as in-person trainings. Hence, online-training might not only be a safer alternative in times of a pandemic, but also an effective means of dissemination of CBT techniques whenever there is a scarcity of experts and long distances to overcome.

Still, there is a lack of studies that directly compare similarly designed in-person and asynchronous concepts of online CBT training courses regarding their acceptability and participants’ satisfaction. In addition to this, the trainer’s effects are often not controlled, despite the possibility that they could heavily influence satisfaction ratings (Ghosh et al., 2012).

Thus, the present study aimed to investigate in a quasi-experimental design whether satisfaction with online CBT training courses is non-inferior in comparison to in-person CBT training courses with the same content (CBT for psychosis), duration of training, comparable audiences, and an identical trainer at five CBT postgraduate training institutes.

## Materials and Methods

### Participants, Recruitment, and Procedure

Participants of the online training courses were enrolled at five different training institutes in Germany (Bielefeld, Bochum, Giessen, Marburg, and Göttingen) between March and April 2020. The training was held by one of the authors of this paper (S. M.). Students were psychologists (M.Sc.) and psychiatrists (second state exam) in the first year of CBT postgraduate training currently employed at inpatient units. The course duration varied between 8 h (Bochum, Giessen, Marburg) and 16 h (Bielefeld, Göttingen). Participants were asked to fill out anonymous paper questionnaires (Göttingen) or to provide their ratings via an online link (all other institutes) after the courses. An additional reminder was sent one week after the course via Email. Items were identical to those questions usually presented at the end of the in-person courses. Depending on the training institute, questions and scales differed slightly in numbers and topics.

In order to obtain data for in-person courses, all training institutes were asked via Email to provide anonymized individual participant data on the trainer’s previous courses, which were held between 2013 and 2019. The duration of courses was identical. Participants were asked to fill out paper questionnaires at the end of the workshop. Participation was not mandatory, and as the data was anonymous, the ethics committee’s approval was not necessary.

#### CBT for Psychosis Online Training

The present asynchronous inverted-classroom online course was run on the Moodle platform. Participants received a fixed time table for the day and met at fixed appointments in six to seven web conferences using Zoom software. They were asked to watch pre-recorded video-lectures presenting theoretical information, patient videos, or audios of interventions between the web conferences. They were also asked to perform exercises by themselves (written reflection on content or questions) or group exercises (training interventions in role-plays via telephone or in web conferences).

The content of the pre-recorded theoretical video lectures was as follows: introduction into CBT; building a positive therapeutic relationship with patients with psychosis; psychopathological symptoms of psychosis and diagnostic criteria; setting motivating therapy goals; psychoeducation and interventions to improve patients’ general mood; interventions to provide psychoeducation on emotions and to train functional and to reduce dysfunctional emotion regulation strategies; interventions to reduce worrying and rumination; interventions to cope with negative emotions such as anger, guilt or anxiety; interventions to reduce negative self-schemata and improve self-esteem; interventions for voices; interventions to challenge delusional beliefs; interventions to reduce risk of relapse. Finally, lectures were provided on prodromal symptoms of psychosis and group interventions for psychosis. Participants also viewed videos of patients with typical positive symptoms of psychosis and listened to an audio recording of a therapeutic intervention. Interventions were selected based on a German manual on CBT for psychosis (Mehl and Lincoln, 2014).

Several exercises supported the training of the intervention: participants had to reflect on previous experiences of psychotic patients to find ways to build a functional therapeutic relationship, to read texts on psychopathology and diagnostic criteria and select correct criteria or a diagnosis, and they were asked to test a mindfulness exercise with another participant. Also, several exercises required training of therapeutic skills: building a positive relationship with patients with psychosis, defining patients’ most important and motivating goals for CBT, implementing and training mindfulness, challenging dysfunctional beliefs on voices, and discussing and challenging delusional beliefs. During web conferences at fixed times (every 2–3 h), all video lectures and exercises were explained in detail, and participants could ask questions. The trainer also asked the group to reflect on typical problems with patients with psychosis and how to solve them, and performed a model role-play (on challenging delusional beliefs) with one participant who played a patient with delusions.

Duration of the online training varied between the CBT institutes between 8 and 16 h; in shorter CBT training, not all pre-recorded video lectures and exercises were provided, but participants had the opportunity to watch video lectures or to practice skills in the 4 weeks following training, as they still had access to the Moodle platform.

#### CBT for Psychosis: In-Person Training

The workshop consisted of the same theoretical lectures as the online workshop. The trainer presented lectures in the classroom, and participants could ask questions. The trainer used the same exercises as in the online workshop; group exercises were performed in separate training rooms.

### Measures

#### Satisfaction and Acceptance Questionnaire

Almost all institutes used the same or a similar questionnaire that included up to eleven items answered on a 6-point-Likert scale (range 1–6) or a 5-point-Likert scale (range 1–5), depending on the institute. Lower scores indicated greater satisfaction. Two items assessed participants’ self-description regarding (1) their *interest in the subject* and (2) whether they had *prior knowledge* of the subject. Nine items measured acceptance and satisfaction with the course: participants were asked whether they were satisfied with (3) the *information content*, (4) the *conception of content*, (5) *didactic presentation*, (6) *room for active participation*, (7) *practical relevance*, (8) *trainer as a suitable role-model*, (9) whether the *working atmosphere* was positive, (10) with their *own commitment*, and (11) *professional relevance* of the workshop (items are presented in Appendix 1). There is some variation of item use and scales, as some institutes did not use all items, but the same items were used in both the on-site and online workshops at the same CBT institutes.

#### Statistical Analyses

All items were carefully analyzed, and answers from different training institutes were aggregated only when identical meaning could be ascertained by two independent raters, resulting in changing numbers of ratings for each item. Since some institutes preferred a 5-point Likert scale and no “6” had been awarded for any item, we interpreted all data along a 5-point Likert scale, as the test statistics we used to assess non-inferiority (explained below) is not influenced by rescaling of Likert-scales (see Kraemer, 2014 for more information).

Usually, mean values and standard deviations for each item are forwarded by the institutes to the trainers for evaluation. This is not considered here as an adequate aggregation method for Likert-like data, as they are in rank-order only (see Jamieson, 2004). Also, with respect to the distribution of the data, Linse states that “most student ratings distributions are skewed, i.e., not normally distributed, with the peak of the distribution above the midpoint of the scale” (Linse, 2017). Our data were expected to take this to the extreme: for each item, the median and modal of the courses’ ratings in the last years held by S. M. had been in the best (i.e., lowest) category almost without exception, rendering a comparative analysis along these aggregation-statistics futile. We also refrained from using a log-transformation of the data in order to obtain a normal distribution since there was no reason to believe that our categorical data followed a log-normal distribution (see Feng et al., 2013 for more information).

#### Analysis of Non-inferiority

Non-inferiority of the online courses vs. in-person courses was analyzed using the averaged Wilcoxon-Mann-Whitney-*U* (WMW-*U*) statistic that was determined for all nine items that assessed participants’ satisfaction with the courses. The averaged WMW-U is a measure of “dominance” of one distribution over the other and can be visualized as the proportion of the area under a ROC curve (AUC), with 0.5 being the value for a pair of mutually non-dominating distributions (see Divine et al., 2018 for more information). AUC can be transformed into the effect size *d*, as explained by Salgado (2018).

Previous studies or meta-analyses that compared online vs. in-person courses used heterogeneous approaches to decide whether courses differed meaningfully regarding their satisfaction and efficacy.

For example, in their meta-analysis comparing effects of inverted-classrooms vs. normal classroom settings on satisfaction, van Alten and colleagues (van Alten et al., 2019) set the *smallest effect size of interest* (SESOI, see Lakens et al., 2018) to *g* = 0.2, but could not provide a definitive answer due to a lack of power (*g* = 0.05; 95% confidence interval (CI): −0.23, 0.32). Nevertheless, they concluded that “students are equally satisfied with the learning environments,” though they could not exclude an effect size of up to *g* = 0.32.

Krogh et al. set *d* = 0.36 as SESOI in their study comparing an online course vs. an in-person course in pediatric basic life support (Krogh et al., 2015). Montassier et al. (2016) even adopted a SESOI of *d* = 0.5 for learning outcomes in online vs. in-person classrooms for the interpretation of ECG data (see Kraemer, 2006 for methods of transformation of effect sizes). The effect size seems large, but on a wide variety of clinical outcomes empirical and theoretical evidence for a SESOI of half a standard deviation (*d* = 0.5) has been provided (Norman et al., 2003).

In their study, Mallonee et al. (2018) reported a significant effect of *d* = 0.35 in satisfaction ratings for online CBT courses in comparison to in-person CBT courses and found the moderators of this difference well worth exploring. They reported that the difference could be largely explained by a shift in participants’ ratings from “very satisfied” to “mostly satisfied.” Experts in student feedback might possibly ignore such a shift in opinion when providing advice for administrator’s evaluation of teaching staff: Linse and colleagues propose a look at the distribution of ratings “as a whole” to check whether “a large percentage of the ratings are clustered at the higher end of the scale” (Linse, 2017), while ignoring “sporadic” ratings at the low end of the scale (Berk, 2013).

Concluding, for our question whether integrating online elements in CBT teaching is an acceptable option even beyond the pandemic crisis, the effect size *d* = 0.35 seems a suitable point of reference. In our context, an effect of this size might be established by a decline of one scale point in ratings of three to four participants of the 18 participants of a typical course that has been transformed from in-person to an online course.

Therefore, we tested whether an effect size larger than *d* = 0.35 between in-person and online course ratings can be rejected. This is the case, whenever the one-sided 95% CI around AUC = 0.5977 does not contain the point-estimator for AUC.

There are several alternative procedures to compute the CI (see Kottas et al., 2014). Among those, the Wald-procedure without continuity correction seems an overall reasonable choice that takes our sample sizes (87 < *n* < 185), highly skewed distributions, a supposedly rather small value of actual AUC and unequal group sizes between 1:2 and 1:3 into account.

## Results

### Enrollment of Participants

At five different CBT institutes, 85 students participated at the online training courses (Bielefeld: *n = 21*; Bochum: *n* = 12; Giessen *n* = 19; Göttingen: *n* = 14; Marburg: *n* = 19). Of this group, a total of 43 participants provided online (*n* = 31; Bielefeld: *n* = 7; Bochum: *n* = 7; Giessen: *n* = 11; Marburg; *n* = 6) or pen-and-paper (Göttingen: *n* = 12) feedback (50.59%).

With regard to in-person courses, we received data of *n* = 142 participants of ten courses at three different training institutes in Germany [two courses in Bielefeld (2018–2019, *n* = 14; *n* = 16), six courses in Göttingen (2013–2018, *n* = 18; *n* = 14; *n* = 11; *n* = 20; *n* = 12 and *n* = 11) and two courses in Giessen (2018; *n* = 13, *n* = 13)].

Sociodemographic data was not assessed in both groups to ensure anonymity. Since all participants were regular aspirants of board certification in CBT at the respective training institutes, a minimum age of 26 and a Master’s degree in psychology or second state exam in medicine was necessary for course enrollment. In a comparable German online study on students in CBT training, the mean age was 30.5 (SD = 5.8: Nübling et al., 2019), and women outnumbered men by a factor of five (86.2% female).

**FIGURE 1:**
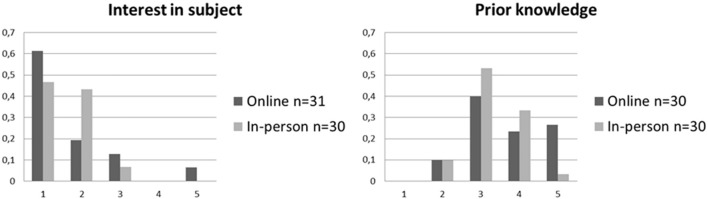
Relative frequ encies of self-reported items of the Satisfaction and acceptance questionnaire for online and in-person courses. 1 = “very much,” 5 = “none.”

On a descriptive level (see Figure 1 and Table 1), with respect to their self-reported *interest in the subject* of the courses, participants in the online courses were comparable with participants in the in-person courses, though more heterogeneous. Participants in the in-person courses rated their *prior knowledge* more positively in comparison to participants in the online courses.

**TABLE 1:** Ratings for online vs. In-person workshops and results of the non-inferiority analysis.

Items		In-person group							Online group						Confidence Interval *d* = 0.35 AUC = 0.5977 *95% CI*	Point estimator $\hat{\text{A}\,U\,C}$
	*n %*	1	2	3	4	5	Median	*n %*	1	2	3	4	5	Median		
*Interest in subject*	30 %	14 46.7	13 43.3	2 6.7	1 3.3	0 0	2	31 %	19 61.3	6 19.4	4 12.9	0 0	2 6.5	1		
*Prior knowledge*	30 %	0 0	3 10	16 53.3	10 33.3	1 3.3	3	30 %	0 0	3 10	12 40	7 23.3	8 26.7	3		
*Information content*	142 %	87 61.3	45 31.7	10 7	0 0	0 0	1	43 %	26 60.5	17 39.5	0 0	0 0	0 0	1	[0.5442, ∞)	0.4901*
*Conception of content*	56 %	30 53.6	20 35.7	6 10.7	0 0	0 0	1	31 %	19 61.3	11 35.5	1 3.2	0 0	0 0	1	[0.5193, ∞)	0.4482*
*Didactic presentation*	142 %	73 51.4	50 35.2	18 12.7	1 0.7	0 0	1	43 %	27 62.8	11 25.6	5 11.6	0 0	0 0	1	[0.5442, ∞)	0.4460*
*Room for active participation*	142 %	85 59.9	46 32.4	8 5.6	3 2.1	0 0	1	43 %	20 46.5	15 34.9	6 14	1 2.3	1 2.3	2	[0.5442, ∞)	0.5834
*Practical relevance*	142 %	95 66.9	38 26.8	8 5.6	1 0.7	0 0	1	43 %	25 58.1	15 34.9	3 7	0 0	0 0	1	[0.5442, ∞)	0.5418*
*Trainer as role model*	141 %	85 60.3	45 31.9	10 7.1	1 0.7	0 0	1	42 %	28 66.7	12 28.6	2 4.8	0 0	0 0	1	[0.5439, ∞)	0.4644*
*Working atmosphere*	142 %	73 51.4	54 38	14 9.9	1 0.7	0 0	1	43 %	23 53.5	12 27.9	7 16.3	1 2.3	0 0	1	[0.5442, ∞)	0.5108*
*Own commitment*	142 %	42 29.6	61 43	34 23.9	4 2.8	1 0.7	2	43 %	12 27.9	21 48.8	7 16.3	2 4.7	1 2.3	2	[0.5442, ∞)	0.4969*
*Professional benefit*	56 %	23 41.1	24 42.9	8 14.3	1 1.8	0 0	2	31 %	11 35.5	15 48.4	5 16.1	0 0	0 0	2	[0.5193, ∞)	0.5222

*H*_0_:*d*0.35, $\hat{A\,U\,C}$ empirical area under the ROC-curve, *CI* = 95% -confidence intervals for $\hat{A\,U\,C}$ under *H_0*. *H_0_ is rejected if $\hat{A\,U\,C}$ is outside *C*I (*p* = 0.05).

**FIGURE 2:**
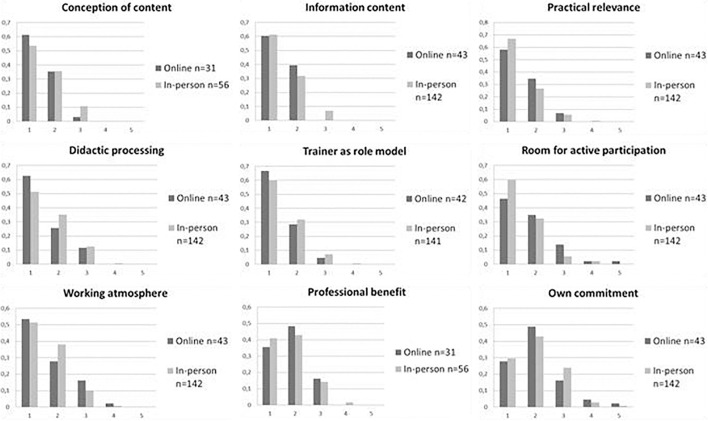
Relative frequencies of items of the Satisfaction and acceptance questionnaire for online and in-person courses. 1 = “very satisfied,” 5 = “not at all satisfied.”

Distributions of satisfaction ratings in both groups are depicted in Figure 2 and Table 1. As expected, all ratings were skewed to the left (with the exception of *own commitment*): a vast majority of participants in both groups rated the courses as satisfying or very satisfying in all items. Table 2 depicts the number and percentage of positive ratings [satisfying (2) or very satisfying (1)] vs. negative ratings (3–5) in both groups. A visual inspection of the data yields similar shapes of the distributions for online and in-person courses.

**TABLE 2:** Descriptive data and percentage of positive vs. neutral/negative ratings in online courses vs. in-person courses.

Items	*n*	In-person courses			Online courses	
		1–2	3–5	n	1–2	3–5
*Information content*	142 %	132 93	10 7	43 %	43 100	0 0
*Conception of content*	56 %	50 89.3	6 10.7	31 %	30 96.8	1 3.2
*Didactic presentation*	142 %	123 86.6	19 13.4	43 %	38 88.4	5 11.6
*Room for active participation*	142 %	131 92.3	11 7.7	43 %	35 81.4	8 18.6
*Practical relevance*	142 %	133 93.7	9 6.3	43 %	40 93	3 7
*Trainer as role model*	141 %	130 92.2	11 7.8	42 %	40 95.2	2 4.8
*Working atmosphere*	142 %	127 89.4	15 10.6	43 %	35 81.4	8 18.6
*Own commitment*	142 %	103 72.5	39 27.5	43 %	33 76.7	10 23.3
*Professional benefit*	56 %	47 83.9	9 16.1	31 %	26 83.9	5 16.1

Results of the non-inferiority test using averaged *U*-values (AUC; see Table 1) revealed that the assumption of inferiority of the online courses could be rejected with regard to the items assessing satisfaction with the *information content, conception of content, didactic presentation*, *satisfaction with the trainer as a suitable role-model, working atmosphere, own commitment*, and *practical relevance*.

The assumption of inferiority of the online courses could not be rejected with regard to the items assessing satisfaction with *room for active participation* and *professional benefit* of the workshop.

## Discussion

An asynchronous, blended, inverted-classroom online learning environment for CBT training (CBT for psychosis) was developed as an emergency solution to training provision during the COVID-19 pandemic. Our study investigated the hypothesis that participants’ ratings regarding acceptance and satisfaction of online CBT courses were non-inferior in comparison to the former in-person courses. For this purpose, we compared ratings of online courses and in-person courses with similar content, length, and trainer with regard to the participants’ satisfaction ratings, using a non-inferiority test (Lakens et al., 2018). Results revealed that participants’ satisfaction with the online courses was not relevantly lower in the categories *information content, conception of content, didactic presentation, satisfaction with the trainer as a suitable role-model, working atmosphere, own commitment*, and *practical relevance* than satisfaction with the in-person training courses. Concerning *room for active participation* and the course’s *professional benefit*, we could not exclude relevantly lower satisfaction ratings in the online courses. Nevertheless, a vast majority of online-participants rated these items as very satisfying or satisfying (81.4%, 83.9%).

With regard to satisfaction with online training courses for CBT therapists, to the best of our knowledge, this is the first study that directly assessed whether an asynchronous online training of identical length, trainer and topic is non-inferior to in-person training.

Our results are built on a study that compared various training courses of some evidence-based CBT techniques (e.g., Prolonged Exposure, CBT for depression) as synchronous online and in-person courses (Mallonee et al., 2018) in a large group of various mental health workers (e.g., therapists, social workers, and pastors). They used a more general measure of satisfaction (one item) and found a statistically significant difference (*d* = 0.35) between the groups. Though being a small-to-moderate effect in the Cohen classification (Cohen, 1992), this difference was not seen as an obstacle for the future use of synchronous online courses by the authors as participants’ ratings were still “satisfied” or “very satisfied” in more than 90% of the cases in both groups.

While synchronous online training cuts geographic connections between trainer and participants, times of learning and teaching remain similar in amount and schedule, posing obstacles to upscaling and dissemination of training e.g., into regions with differing time zones and a lack of suitable experts. Going one step further, we aimed to investigate whether loosening the time-bonds by adding asynchronous elements could still establish equal satisfaction.

Our positive answer for most of the items is in line with a meta-analysis that found no differences in satisfaction rates between in-person teaching in inverted and traditional classrooms (van Alten et al., 2019) in higher education and medical training (Chen et al., 2018).

However, we could not exclude the online training’s inferiority regarding the items *room for active participation* and *professional benefit* even though the number and content of exercises were equal in both online and in-person courses. With respect to the active participation of the audience, we assume that the differences are related to the reception of the pre-recorded lectures: participants could not pose their questions spontaneously during the lecture but were required to write them down. Posed later, the context of the question might not have been at the center of attention for the other participants. This might have framed the question as a special issue of the inquirer, leading to less engagement of the rest of the group in spontaneously emerging discussions of practical cases.

In addition, emerging discussions in web conferences are hindered by the fact that social cues such as eye gaze and body gestures cannot be used in an online setting to determine the audience’s degree of interest and adapt speech content towards it. The lack of these implicit gestures also leads to difficulties in signaling turn-taking in conversations (see Rossano, 2012 for more information). Furthermore, joining into the discussion is often hindered by the need to activate microphones. The resulting toll on participants’ concentration has been named “Zoom fatigue” (Wiederhold, 2020). From the trainer’s point of view, this might have impeded her from telling stories from everyday practice, which might explain the reduced professional benefit.

Summing up, the asynchronous, online blended learning solution had some practical advantages at relatively small costs in certain dimensions of satisfaction. Whenever there is a scarcity of shared room, shared time or specialized trainers on the spot, online CBT courses seem to be a feasible and acceptable solution.

### Limitations

First, we are not able to report demographic data of the participants, due to the fact that our study was part of the regular CBT training in Germany in small groups (≤18): usually demographic data are not gained to prevent de-anonymization.

In addition, we collected the data via both an online form and pen-and-pencil questionnaires. Several studies suggest that online assessments are as representative as paper-and-pencil evaluations and yield similar results (Spooren et al., 2013). Nevertheless, in the online courses, drop-out rate was more pronounced (41%) in comparison to the in-person courses where we had data from all participants. Although drop-out rates are typically higher in online satisfaction assessments (Ardalan et al., 2007), it is possible that we only included participants who were more satisfied with the online courses. This also led to an unequal sample size in our analysis, however, we remedied this problem by using a robust computation of the confidence intervals, as recommended by Kottas et al. (2014).

Participants were not randomized to the CBT training conditions; thus, we are not able to exclude a cohort effect. Furthermore, both 6-point-Likert scales and 5-point-Likert scales were used and then combined for our analysis, which is not ideal, but did not affect the non-parametric analyses (also, ratings of 6 were not awarded on the 6-point Likert scales).

As our study was part of the regular CBT training for therapists in Germany, no objective measures of the degree of gained knowledge was included. Nevertheless, student’s satisfaction ratings are known to be reliable and valid (Linse, 2017), although the correlation between students’ satisfaction ratings and their learning outcome is small (Abrami, 2001; Eizler, 2002). However, in contrast to students in general, our participants had at least 6 years of previous experience in higher education and can thus be considered experienced in the evaluation of didactic presentations. Since the workshops prepared our participants for an important exam, it is plausible that the amount of gained knowledge influenced their acceptance ratings. Furthermore, no objective evaluation of the skills learned during the training was assessed by the trainer. Also, there were no data on qualitative satisfaction ratings available that would be helpful in evaluating students’ subjective experience.

Because of the satisfaction ratings’ subjective nature, the emotional background of the online courses is worth considering. We delivered the asynchronous online inverted-classroom courses during the COVID-19 pandemic as an emergency solution. Thus, participants were well aware that the trainer had voluntarily increased her effort at short notice to transfer the content into online courses. Satisfaction ratings of the participants might have been influenced by their gratitude for being able to continue their CBT training.

Furthermore, satisfaction ratings might also depend on the courses’ subject (CBT for psychosis) that might be considered more interesting than other topics. Also, some CBT courses might be more easily transferred to an online format in comparison to CBT courses that focus more on training of interventions (e.g., imagination techniques, chair work). Thus, the generalizability on other topics of CBT training is unclear.

### Implications for Future Studies

With regard to the special situation of the pandemic discussed above, a first step would be to replicate our study in a non-pandemic situation as a pre-registered randomized-controlled study. It is also important in future studies to assess whether asynchronous online courses are non-inferior in comparison to synchronous online courses regarding satisfaction ratings and to use additional outcome criteria, e.g., to include assessments of gained knowledge, evaluation of gained knowledge by the trainer and to test participants on their newly acquired therapeutic skills.

In order to gain realistic, less pandemic-dependent data on satisfaction ratings, perceived advantages and shortcomings of the online setting can be assessed by the so-called “willingness to pay” paradigm (WTP) in economics. In this setting, scientists or CBT institutes might offer an online-course at the same time as the regular in-person course. Students would have to place a bid for online-participation, which can be either positive (they signal their willingness to pay an additional fee for the perceived advantages), or negative (they signal the amount of reduction they expect for the perceived restricted service). The online course is then sold to the half of the group with the highest bids at the price of the lowest bid in that half of the group (see Breidert et al., 2006 for a review of WTP-assessments).

### Implications for CBT Training Post the COVID-19 Pandemic

Asynchronous online courses offer several advantages with respect to convenience and costs. Since candidates are usually employed in a full-time position at different locations and often have caring duties, a more individual schedule and no need for traveling might be appreciated by participants. Without the need for traveling and staying overnight, highly qualified trainers might be easier to find. Of course, this comes at the price of a more individualized learning experience, e.g., no informal meetings at the coffee station and diminished sense of belonging to a community of learners.

There are a number of potential solutions in order to give participants more room for active participation in online courses. One solution is to start the course with short “break out” exercise sessions in smaller groups. Participants often have less problems to engage in these smaller groups, as it is less important to mute their microphone when not talking (to prevent white noise). In these smaller groups, it is also easier to help each other to get used to the software.

In order to encourage participants to ask more questions, the trainer could start each web-conference with a short visual summary of the video lectures the participants were required to watch between the web conferences and could share the slides of her presentation. Also, she could motivate participants to ask questions either verbally or by writing down the question in the group chat.

In addition, participants were often interested in the trainer performing a therapeutic intervention role-play with a student role-playing as a client. These live role-plays in large groups can be improved if all students except the participants of the role-plays deactivate their cameras in order to enhance visibility of the participants. In addition, students can write down additional questions or suggest interventions for the trainer to read during the exercise in the group chat. Obviously, it is beneficial afterward to train the interventions in small groups in break out rooms, supported by the trainer. Also, playful quizzes or tests at the end of the courses could positively influence the effectiveness and attractiveness of online courses (Spanjers et al., 2015).

## Conclusion

The present study yields some interesting results: it provides first evidence that ratings of satisfaction of an online asynchronous CBT training are not inferior to ratings of an in-person training provided by the same training person at the same training institutes regarding various dimensions of satisfaction (information content, conception of content, didactic presentation, satisfaction with the trainer as suitable role-model, working atmosphere, own commitment, and usefulness for own practice). The results also suggest that some differences between online and in-person courses could not be excluded with regard to active participation and practical orientation.

Our results indicate that integrating online elements in CBT teaching is an acceptable option even beyond the pandemic crisis.

## Data Availability Statement

The raw data supporting the conclusions of this article will be made available by the authors, without undue reservation.

## Ethics Statement

Ethical review and approval was not required for the study on human participants in accordance with the local legislation and institutional requirements. Written informed consent for participation was not required for this study in accordance with the national legislation and the institutional requirements.

## Author Contributions

DS performed the statistical analyses. All authors conceived the study, planned the trial’s design, wrote the manuscript, read, and approved the final manuscript.

## Conflict of Interest

The authors declare that the research was conducted in the absence of any commercial or financial relationships that could be construed as a potential conflict of interest.

## Publisher’s Note

All claims expressed in this article are solely those of the authors and do not necessarily represent those of their affiliated organizations, or those of the publisher, the editors and the reviewers. Any product that may be evaluated in this article, or claim that may be made by its manufacturer, is not guaranteed or endorsed by the publisher.

## Appendix 1

Items of the acceptance questionnaire (translated by the authors)

Please rate how…

(1) strongly you were interested in the subject of the workshop?
(2) broad was your prior knowledge?

Please rate your satisfaction with…

(3) the information content of the workshop?
(4) the conception of content of the workshop?
(5) didactic presentation?
(6) room for active participation?
(7) practical relevance?
(8) trainer as suitable role model?
(9) working atmosphere?
(10) own commitment during the workshop?
(11) professional benefit of the workshop?

Fragebogen zur Qualitätssicherung der theoretischen Ausbildung (QS1)

Bitte geben Sie an, wie groß…

(1) Ihr Interesse am Thema war?
(2) Ihre Vorkenntnisse zu dem Thema waren?

Bitte stufen Sie ein, wie zufrieden Sie sind mit…

(3) dem Informationsgehalt?
(4) der inhaltlichen Konzeption?
(5) der didaktischen Präsentation?
(6) der Möglichkeit zur aktiven Beteiligung?
(7) dem Praxisbezug?
(8) dem Referenten/der Referentin als Rollenmodell?
(9) der Arbeitsatmosphäre?
(10) mit dem eigenen Engagement während der Veranstaltung?
(11) dem Nutzen für die eigene Tätigkeit?
